# Focused ultrasound-enhanced intranasal brain delivery of brain-derived neurotrophic factor

**DOI:** 10.1038/srep28599

**Published:** 2016-06-27

**Authors:** Hong Chen, Georgiana Zong Xin Yang, Hoheteberhan Getachew, Camilo Acosta, Carlos Sierra Sánchez, Elisa E. Konofagou

**Affiliations:** 1 Department of Biomedical Engineering, Columbia University, New York, New York, USA; 2 Department of Radiology, Columbia University, New York, New York, USA

## Abstract

The objective of this study was to unveil the potential mechanism of focused ultrasound (FUS)-enhanced intranasal (IN) brain drug delivery and assess its feasibility in the delivery of therapeutic molecules. Delivery outcomes of fluorescently-labeled dextrans to mouse brains by IN administration either before or after FUS sonication were compared to evaluate whether FUS enhances IN delivery by active pumping or passive diffusion. Fluorescence imaging of brain slices found that IN administration followed by FUS sonication achieved significantly higher delivery than IN administration only, while pre-treatment by FUS sonication followed by IN administration was not significantly different from IN administration only. Brain-derived neurotrophic factor (BDNF), a promising neurotrophic factor for the treatment of many central nervous system diseases, was delivered by IN followed by FUS to demonstrate the feasibility of this technique and compared with the established FUS technique where drugs are injected intravenously. Immunohistochemistry staining of BDNF revealed that FUS-enhanced IN delivery achieved similar locally enhanced delivery as the established FUS technique. This study suggested that FUS enhances IN brain drug delivery by FUS-induced active pumping of the drug and demonstrated that FUS-enhanced IN delivery is a promising technique for noninvasive and localized delivery of therapeutic molecules to the brain.

Brain drug delivery for the treatment of a wide variety of central nervous system (CNS) diseases is hampered by the blood-brain barrier (BBB)^1^. Intranasal (IN) brain drug delivery is a noninvasive approach for transporting therapeutics from the nose directly into the CNS through the unique olfactory and trigeminal neural pathways that connect the nasal mucosa with the perivascular spaces within the CNS^2^. A variety of therapeutic agents, including peptides, proteins, gene vectors, and stem cells have been delivered to the brain through the IN route^2^. This route has unique advantages: bypassing BBB, drug administration is noninvasive and comfortable, delivery to the CNS is relatively rapid, dosages are repeatable, no modification of the drugs is required, systemic exposure is minimized, and the brain drug level is two to three orders of magnitude higher using IN compared to intravenous (IV) injection^3^,^4^. However, the application of this noninvasive and convenient brain drug delivery technique is hampered by two main challenges: low drug delivery efficiency due to poor nasal mucus permeability and widespread drug delivery in the brain, which is unfavorable as neurological diseases do not generally affect the brain in a global manner.

Brain-derived neurotrophic factor (BDNF), the most widely expressed and studied neurotrophin in the mammalian brain, has emerged as a promising agent for the treatment of many CNS diseases, such as Alzheimer’s Disease, Parkinson’s disease, Huntington’s disease, and stroke^5^. However, a significant challenge to its clinical usages is the difficulty associated with its delivery to the brain parenchyma^6^,^7^. As a 27-kDa protein dimer, BDNF has minimal BBB penetrability^8^, a short half-life in blood (0.92 min)^9^, and a poor pharmacokinetic profile, making its delivery to the brain difficult. Their access to the CNS is restricted by rapid enzymatic inactivation, multiple clearance processes, potential immunogenicity and sequestration by binding proteins and other components of the blood and peripheral tissues^8^. Some *in vivo* strategies for delivering BDNF to the CNS include direct injection into the brain and infusion pump-mediated delivery^3^. Unfortunately, these methods are invasive and limited by diffusion restrictions. Systemic IV delivery is not practical because these large neurotrophic proteins do not efficiently cross the BBB and have high systemic side-effects. A cluster of acute systemic symptoms after IV injection of BDNF in patients has been reported: dyspnea, flushing, throat tightness, nausea, rigors and tachycardia^10^. Therefore, alternative delivery methods are needed to realize the clinical promise of BDNF and other similar neuroprotective drugs.

Our previous study proposed a novel, noninvasive drug delivery strategy: focused ultrasound (FUS)-enhanced IN delivery^11^. The nose-brain pathways along the olfactory and trigeminal nerves that innervate the nasal passages are involved in the transport of drugs directly from the nose to the brain. After drugs reach the brain entry points they distribute to the whole brain along the cerebral perivascular spaces – thin annular regions surrounding cortical blood vessels – and may be propelled through perivascular spaces by heartbeat-driven pulsations of blood vessel walls, called “perivascular pump effect”^12^,^13^. The FUS technique uses extracorporeal generated ultrasound beams that can penetrate through the skull and focus onto a small focal region (on the order of millimeters) to activate intravenously injected microbubbles within the FUS targeted region. FUS combined with microbubbles has been used as an effective and safe method for noninvasive, localized, and transient BBB opening^14^, so IV-injected drugs can be delivered across the BBB to the brain parenchyma^15^. The same mechanical forces generated by microbubbles on the vessel wall for BBB opening also cause the expansion and contraction of the perivascular spaces^16^. We hereby call this the “microbubble pump effect”. The similarity between the microbubble pump effect and the perivascular pump effect led us to hypothesize that the microbubble pump effect contributes to FUS-enhanced IN brain drug delivery.

To assess whether the microbubble pump effect contributes to FUS-enhanced IN brain drug delivery, we compared two treatment protocols: (1) pretreatment of mouse brains by FUS in combination with microbubbles followed by IN administration (FUS + IN); (2) IN administration immediately followed by FUS treatment (IN + FUS). The microbubble pump effect could only contribute to the drug delivery outcome in protocol (2) where drugs were present in the perivascular space when FUS was applied. Fluorescently-labeled dextrans with molecular weight (MW = 40 kDa) similar to BDNF (MW = 27 kDa) were used as the model drug. With the understanding of the potential physical mechanism of FUS-enhanced IN brain drug delivery, we demonstrated the feasibility of this technique for the delivery of BDNF and compared with the established FUS technique where drugs are injected through IV (IV + FUS).

## Results

**Figure 1:**
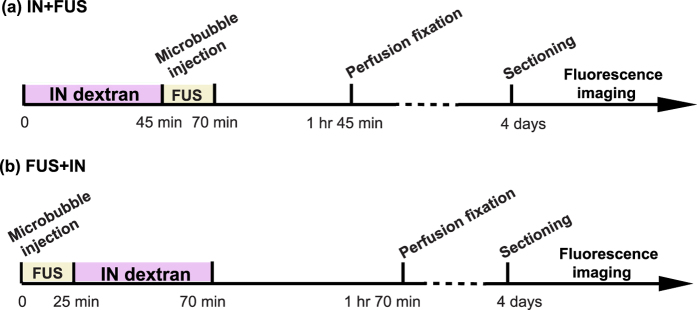
Experimental timeline for (**a**) IN-administration followed by FUS sonication (IN + FUS) and (**b**) pretreatment by FUS followed by IN administration (FUS + IN).

We first compared the two treatment protocols: (1) IN administration followed by FUS sonication (IN + FUS) and (2) pretreatment by FUS followed by IN administration of dextrans (FUS + IN) (Fig. 1). Fourteen wild-type mice were equally divided into two groups. FUS sonication at the left caudate putamen (CPu) was performed immediately after intravenous injection of microbubbles. The right side caudate putamen was used as the control for IN administration only. A total of 2 mg of fluorescently-labeled 40 kDa dextrans were administered into the nose of each mouse in 3 μL drops by alternating nostrils every 2 minutes either before or after FUS sonication. For both groups, mice were sacrificed at 1 hr after IN administration. Then mouse brains underwent perfusion, fixation, frozen sectioning, and fluorescence imaging.

**Figure 2:**
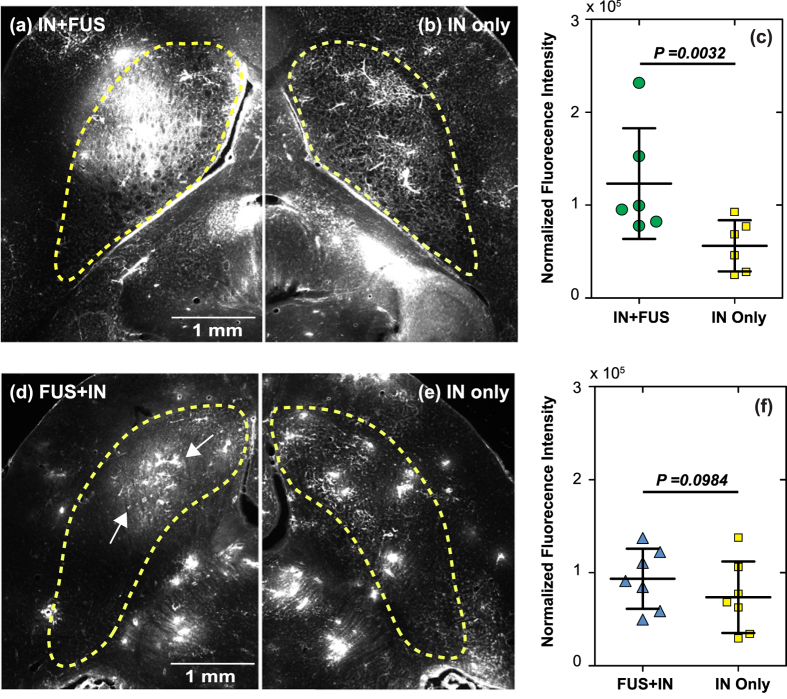
Compare two protocols for focused ultrasound-enhanced intranasal brain drug delivery. Fluorescence images of the left caudate putamen treated by (**a**) IN-administration followed by FUS sonication (IN + FUS) or (**d**) pretreatment by FUS followed by IN administration (FUS + IN). (**b**,**e**) The corresponding right caudate putamen from the same mouse was used as control for IN only. Dash lines highlight the caudate putamen regions. (**c**,**f**) quantitative fluorescence intensity analysis of the two treatment protocols. FUS significantly enhanced IN delivery efficiency when drugs were administered through IN before FUS sonication. There was no statistically significant enhancement when drugs were administered after IN; however, slightly enhanced delivery at the FUS targeted region was observed in the case shown in (**d**) at the FUS targeted location (the region between the two arrows).

**Figure 3:**
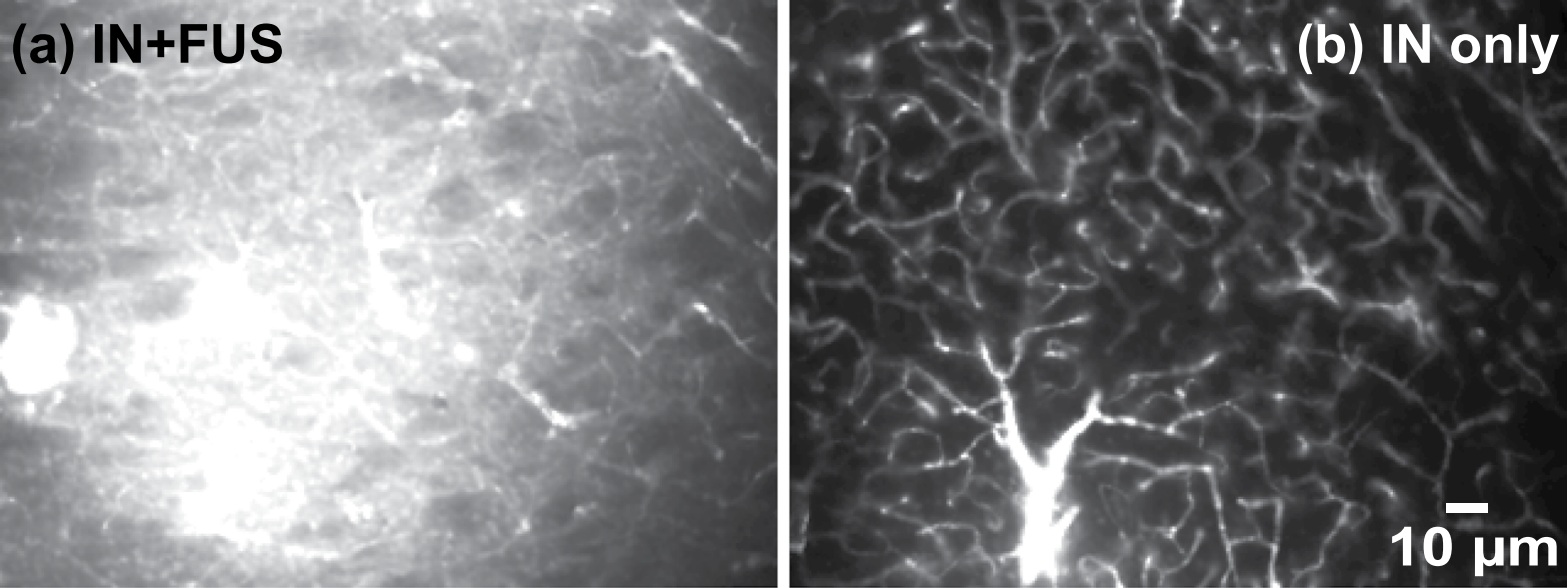
Higher magnification images present the locally-enhanced delivery effect of FUS on IN drug delivery. (**a**) FUS sonication enhanced dextran penetration and diffusion at the focused ultrasound targeted region. (**b**) Without FUS sonication, the fluorescently-labeled dextrans were confined within the perivascular space.

Figure 2a,d present representative fluorescence images of the left CPu treated by IN + FUS and FUS + IN, respectively. Figure 2b,e display the corresponding right side of the CPu without FUS treatment, which served as IN only control. In these images, bright pixels represent fluorescently-labeled dextrans that were delivered to the brain tissue. Figure 1b,e confirmed that 40 kDa dextrans could be delivered to the brain through the IN route, bypassing the BBB. IN administration followed by FUS showed enhanced dextran delivery at the FUS targeted location. Quantification of the fluorescence intensity of the whole group shows a statistically significant (*p* < 0.05) enhancement in fluorescence intensity with IN + FUS treatment as compared with IN only (Fig. 2c). However, there was no statistically significant (*p* > 0.05) difference between FUS + IN and the contralateral IN only control (Fig. 2e), although slightly enhanced delivery at the FUS targeted region was observed in some cases (Fig. 2d). Figure 3b shows a higher magnification image obtained from the IN-only side, which shows that the cerebral vascular network was highlighted by the IN-administered dextrans in the perivascular space. We know the dextrans were not inside the lumen of the blood vessels because all the dextrans in the blood were flushed away by transcranial perfusion. It confirms that IN-administered dextrans are distributed throughout the entire brain along the vascular network and confined within the perivascular space. Figure 3a is a higher magnification image obtained from the IN + FUS side, showing diffusion and penetration of the dextrans into the brain tissue at the FUS targeted location.

**Figure 4:**
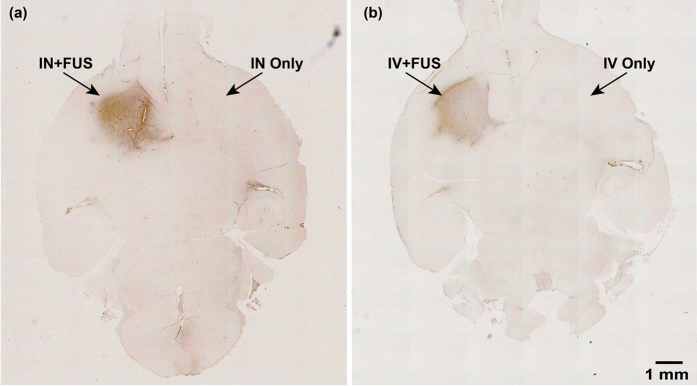
BDNF delivery by FUS-enhanced intranasal drug delivery (IN + FUS) and FUS combined with IV drug injection (IV + FUS). (**a**,**b**) are the immunohistochemistry staining of the whole brain horizontal section from mice in the IN + FUS and IV + FUS groups, respectively. FUS was targeted at the left caudate putamen. Locally enhanced delivery was achieved for BDNF administered either through IN or IV.

**Figure 5:**
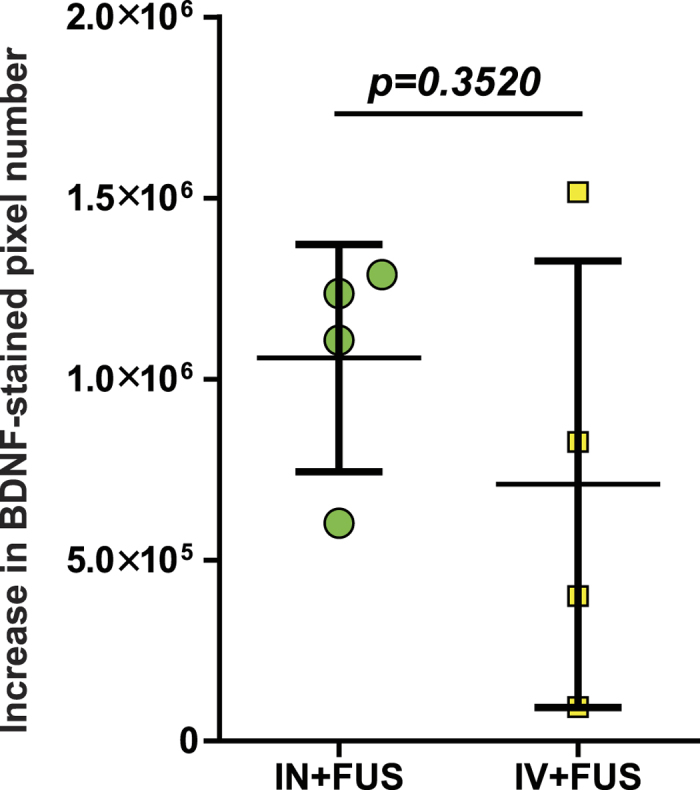
Quantification of BDNF delivery. No significant difference in the enhancement of BDNF delivery when comparing the increase in BDNF-stained pixel numbers of FUS treated caudate putamen and the contralateral nontreated control side.

After confirming that the IN administration followed by FUS sonication can lead to significantly enhanced brain drug delivery, we evaluated the feasibility of this technique for the delivery of BDNF and compared the delivery outcomes with the established FUS sonication technique that is coupled with IV drug injection (IV + FUS). Wild-type mice (n = 8) were divided into two groups. One group (n = 4) was administered 500 μg of BDNF through the nose followed by FUS treatment. The other group (n = 4) was injected with the same amount of BDNF through the tail vein followed by FUS treatment. To ensure safe delivery without causing any tissue damage, we decreased the FUS pulse length from 10,000 cycles as used in the previous study^11^ to 1,000 cycles. After treatment, the mouse brains were prepared for immunohistochemical (IHC) staining. The distribution of BDNF was determined by assessing the distribution of brown 3,3′-Diaminobenzidine (DAB) chromogenic substrate precipitated during IHC staining of BDNF. BDNF delivery was quantified by calculating the increase of brown color pixels within the FUS sonicated CPu region over the contralateral control side. Figure 4a,b are bright field images of mouse whole brain horizontal sections acquired by a microscope with stitching function. Figure 4a was obtained from a mouse treated by IN + FUS on the left CPu while the right CPu served as the control for IN only. Figure 4b was captured from a mouse treated by IV + FUS on the left CPu while the right side served as control for IV only. Locally enhanced delivery of BDNF was observed in both IN + FUS- and IV + FUS- treated CPu compared with the corresponding controls. The number of pixels with dark brown staining was quantified. The difference in the number of stained pixels in the FUS-treated CPu compared with the non-treated side was calculated for each mouse to evaluate the enhancement of BDNF delivery by FUS. No significant difference was found between the IN + FUS and IV + FUS treatment groups (Fig. 5).

**Figure 6:**
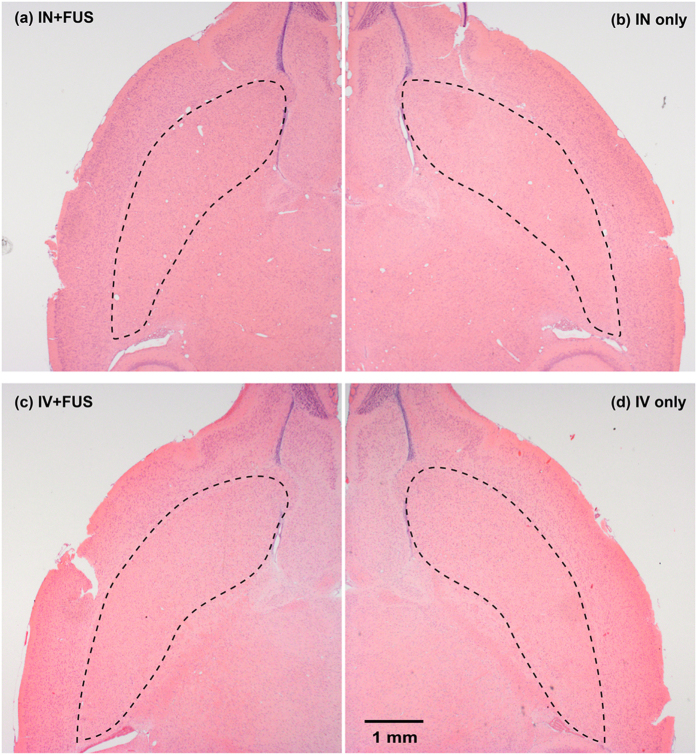
Histological examinations by hematoxylin and eosin (H&E). No tissue damage was observed in the IN + FUS and IV + FUS groups. Representative H&E stained images are shown here. (**a**,**c**) FUS sonicated left caudate putamen and (**b**,**d**) corresponding nonsonicated right caudate putamen.

Sections adjacent to those stained for BDNF for both IN + FUS and IV + FUS groups were subjected to routine hematoxylin and eosin (H&E) staining. Of all the mice analyzed, no mouse exhibited any characteristics of edema or vascular damage. Figure 6 shows H&E-stained brain horizontal sections from the same mice as shown in Fig. 4. Comparing the FUS-treated (left) CPu with the contralateral (right) control side, no histological tissue damage was observed.

## Discussion

Treatment of CNS diseases is hampered by the BBB. IN brain drug delivery is a promising technique to circumvent the BBB; however, its broad application is limited by its low delivery efficiency and non-localized aspect. This study demonstrated that IN administration followed by FUS sonication can significantly enhance IN brain drug delivery at the FUS targeted brain locations and this technique can be used for noninvasive and localized delivery of BDNF.

IN administered drugs transport to the brain through the olfactory and trigeminal nerves. After drug reaches brain entry points they distribute to the whole brain in the cerebral perivascular spaces potentially by the perivascular pump effect: the expansion and contraction of the vessel with the cardiac cycle^12^,^13^ propel the drugs to move forward in the perivascular spaces. Microbubbles expand and contract when activated by the FUS waves, a phenomenon known as acoustic cavitation. The oscillating microbubbles are thought to stretch on the blood vessel walls to induce BBB opening^17^. Previous studies using ultra-high speed microphotography showed that microbubble oscillation in microvessels can push and pull on the vessel walls as well as the perivascular space^16^. The expansion and contraction of the perivascular spaces may induce bulk flow, leading to enhanced convective transport and increased penetration of drugs into the brain tissue. As drugs are transported away from the perivascular space to the parenchyma at the FUS sonicated region, the concentration of drugs in the perivascular space within the targeted region would become lower. Then, driven by the concentration gradient, more drugs in the perivascular space outside the FUS targeted region may diffuse into the targeted region. In this way, the microbubbles may continuously “pump” drugs into the brain tissue, leading to enhanced delivery of IN-administered drugs at the FUS targeted region.

We compared the delivery outcomes of two treatment strategies and found that IN followed by FUS showed significant increase in drug delivery when compared with IN only and pretreatment by FUS followed by IN did not significantly enhance IN delivery efficiency (Fig. 2). When drugs were administered before FUS sonication, the microbubble pump effect contributed to the enhanced drug delivery; however, when drugs were administered after FUS sonication, the microbubble pump effect could not affect the drug delivery outcome as the drugs were not in the perivascular space when microbubbles were activated by the FUS. Figure 3 clearly showed that FUS greatly enhanced the delivery of IN-administered dextrans to the brain parenchyma, while without FUS sonication IN-administered dextrans were confined within the perivascular space. CPu was selected as the targeted region in this study. The targeted region can be controlled by adjusting the location of the FUS focal region. While, IN-administered drug passively diffused into tissue in the FUS + IN group. Note that the slightly enhanced delivery at the FUS-targeted region was observed in Fig. 2d, which indicates that FUS can also passively improve drug diffusion. However, the enhancement effect was not strong enough to induce significant increase in drug delivery when compared against the IN-only control group. Overall, these findings strongly indicate that the microbubble pump effect may play an important role in FUS-enhanced IN brain delivery. We note that this study only provided indirect evidence that supports the microbubble pump effect hypothesis and future studies demonstrating the effect are required to further validate this hypothesis.

With the understanding of the potential physical mechanism for FUS-enhanced IN brain drug delivery, we demonstrated the feasibility of this technique for the delivery of BDNF. Although promising for the treatment of CNS diseases, neurotrophic factors have been proven challenging as pharmacological agents, because of their short half-life in the blood stream (<1 min), minimal BBB-penetration, and high systemic side effects^8^. BDNF can be delivered to the required site of action through invasive procedures which involve injection of catheters or implantable pumps into the brain. Noninvasive delivery of BDNF by IN administration has been explored before^18^. IN administration is simple, allowing rapid drug delivery to the brain with minimal systemic exposure of the drug^8^. However, drug delivery selectively through the nose to the desired brain sites at the intended dose remains challenging. Our group’s previous work has shown that FUS-induced BBB opening is feasible for noninvasive and targeted delivery of intravenously injected BDNF^19^. Consistent with our previous work, the present study confirmed that the established FUS + IV technique is a reliable strategy for BDNF delivery to the brain. However, systemic IV delivery at specific doses in humans has been shown to incur systemic side-effects such as dyspnea, flushing, throat tightness, nausea, rigors and tachycardia^10^. Using IHC staining of BDNF, we showed that IN + FUS achieved similar targeted BDNF delivery to IV + FUS (Figs 4 and 5). Enhanced drug delivery at the FUS-targeted location was achieved with the IN + FUS technique, which overcame the main challenges of IN delivery. Histological analysis found no tissue damage associated with the FUS treatment (Fig. 6). Moreover, the nose-to-brain transport restricts extensive systemic circulation such as that incurred in IV injection, reducing the risk of systemic side effects and hepatic/renal clearing^20^. Although the nasal anatomy and physiology of humans are different from rodents, studies in humans have demonstrated that drugs can directly reach the brain following IN administration and the nose-to-brain transportation mechanism is similar to that of the rodents^21^,^22^. Our preliminary study suggests that IN + FUS is a promising approach for BDNF delivery. IN + FUS may become a viable alternative for FUS + IV in the delivery of therapeutics that have beneficial effects within the CNS, but short half-lives in the blood and/or high systemic side effects, such as neurotrophic factors, neuropeptides, and hormones.

There are several limitations of this study. First, the number of pixels with dark brown staining was quantified to compare the two drug delivery techniques. Different from the fluorescence markers, the concentration of the DAB reaction product is not linearly proportional to its optical density. Therefore, the DAB intensity is not reliable for evaluating the amount of antigen expression. Additional studies are needed to quantify the amount of BDNF delivered by IN + FUS and IV + FUS using techniques such as high performance liquid chromatography (HPLC) or enzyme-linked immunosorbent assay (ELISA). Second, further studies are needed to quantify the drug amount in the blood, cerebral spinal fluid, and other organs to fully evaluate drug distribution by IN + FUS treatment. Third, safety of the FUS technique was demonstrated by histological staining of the brain tissue, while olfactory mucosa was not evaluated. Future studies will establish the complete safety profile of this technique.

## Material and Methods

### Animals

This study was carried out in strict accordance with the recommendations in the Guide for the Care and Use of Laboratory Animals of the National Institutes of Health. The protocol was approved by the Columbia University Institutional Animal Care and Use Committee. All surgery was performed under isoflurane anesthesia, and all efforts were made to minimize animal suffering. The animal body temperature was maintained using a heating pad.

### Microbubbles

Microbubbles comprised of a 90 mol% 1,2-distearoyl-sn-glycero-3-phosphocholine (DSPC) and 10 mol% 1,2-distearoyl-sn-glycero-3-phosphoethanolamine-N-[methoxy(polyethyleneglycol)2000] (DSPE-PEG2000) (Avanti Polar Lipids, Alabaster, AL, USA) lipid-shell and a perfluorobutane (FluoroMed, Round Rock, TX, USA) gas-core were manufactured in-house. Size-selected microbubbles with a median diameter of 4–5 μm were isolated from a poly-dispersed microbubble distribution using a differential centrifugation method [2]. Their size distributions and concentrations were determined by a particle counter (Multisizer III, Beckman Coulter Inc., Opa Locka, FL, USA). Before each injection, their concentrations were diluted using sterile saline to a final concentration of approximately 8 × 10^8^ number of microbubbles per mL.

### IN administration

Fluorescently labeled dextran and BDNF were administered to mice through the nasal route following a procedure used in our previous publication^11^. They were dissolved in saline for a final concentration of 20.8 ug/uL. The anaesthetized mice were placed supine (on their backs) with the head horizontally aligned with the rest of the body. A micropipette was used to intranasally administer 3 μL drops of BDNF solution to alternating nostril every 2 minutes. Drops were placed at the opening of the nostril, allowing the animal to snort each drop into the nasal cavity. A total of 24 μL solution was delivered over a course of 34 min.

### Fluorescently labeled dextran delivery

A total of 14 male C57BL/6 mice (20–25 g in weight; Harlan Laboratories, Indianapolis, IN) were used for fluorescently-labeled dextran delivery. They were divided into the following two experimental groups with n = 7 for each group: (1) FUS + IN treatment group: FUS treatment on the left side of CPu followed by IN administration of 40 KDa dextrans; (2) IN + FUS treatment group: IN administration of dextrans followed by FUS treatment on the left side of the caudate putamen. The contralateral right CPu was not sonicated and used as the control for IN only.

The mice were sonicated using an experimental setup described before^11^. Before FUS sonication, each mouse was positioned prone with its head immobilized by a stereotaxic frame (David Kopf Instruments, Tujunga, CA, USA). The FUS transducer was moved 2 mm lateral of the sagittal suture and 6 mm anterior of the lambdoid suture to target the CPu. Freshly diluted microbubble suspension (30 μL) was administered intravenously via the tail vein prior to each sonication. Immediately after microbubble injection (~5 s), FUS (center frequency: 1.5 MHz; peak-negative pressure: 0.45 MPa; pulse length: 10,000 cycles; pulse repetition frequency: 5 Hz; duration: 1 min) was applied transcranially to the left CPu.

### BDNF delivery

A total of 8 male C57BL/6 mice (20–25 g in weight; Harlan Laboratories, Indianapolis, IN) were used for BDNF delivery. They were divided equally to two groups: (1) IN + FUS and (2) IV + FUS.

For the IN + FUS group, BDNF was intranasal administered followed by FUS sonication at the left CPu in the presence of systemically injected microbubbles. Same acoustic parameters were used as the dextran delivery with the only difference that the pulse length decreased from 10,000 cycles to 1,000 cycles to ensure the safety of the FUS sonication. For the IV + FUS treatment group, microbubbles were co-injected with BDNF. Immediately after injection (~5 s), FUS was applied to the left caudate putamen. The non-sonicated right CP served as control for IN administration only (group 1) or IV injection only (group 2).

### Brain imaging

For all the mice used in the current study, there was a 1-h wait period after dextrans or BDNF administration to allow drugs diffuse into the brain parenchyma. At the end of the allotted time, all mice were sacrificed by transcardial perfusion. For dextran delivery, *ex vivo* mouse brains were frozen and sectioned to 60-μm thick horizontal sections. For BDNF delivery, the mouse brains were processed and prepared for paraffin (6 μm thick) sectioning. The paraffin sections were either stained for BDNF by IHC staining for later use in quantifying BDNF delivery outcomes or stained by hematoxylin and eosin (H&E) staining for whole brain histological examinations [1].

An antibody against human BDNF (Anti-BDNF antibody [35928.11], Abcam, Cambridge, UK) was used for IHC staining of BDNF. A standard protocol for deparaffinization, hydration, and blocking was then followed. The blocking serum was then removed and the primary antibody anti-BDNF (1:100 rabbit) was added. After washing, ABC reagent (A 1:50, B 1:50 in PBS 30 minutes before being mixed) was added for 30 minutes at room temperature. Slides were again washed. DAB solution (DAKO North America, Carpinteria, CA, USA) was then added to each section to promote development of brown stain. Slides were dehydrated and mounted with coverslips.

### Imaging and quantification

Fluorescently-labeled dextrans were imaged by a fluorescence microscopy. IHC stained BDNF slides were imaged using a light microscope and were white corrected. Algorithms written in Matlab were implemented to perform image quantification. A circular region of interest (ROI, diameter = 1.2 mm) was manually aligned to the targeted and control sections of the CPu. For dextran quantification, the spatial average fluorescence intensities were calculated within each ROI and normalized by the background level for comparison among animals. BDNF delivery outcomes were determined by quantifying the DAB stained pixels. This algorithm calculated the numbers of pixels with intensities higher than the background. The difference in pixel numbers between the treated side and the control was calculated for each mouse and used to represent the drug delivery enhancement effect of the treatment.

### Statistical Analysis

An unpaired two-tailed Student’s t-test using GraphPad Prism (Version 5.01, La Jolla, CA, USA) was used to compare between groups. A *p* value of 0.05 was considered to represent a significant difference in all the analyses. All data were expressed as mean ± standard deviation.

## Additional Information

**How to cite this article**: Chen, H. *et al*. Focused ultrasound-enhanced intranasal brain delivery of brain-derived neurotrophic factor. *Sci. Rep.* **6**, 28599; doi: 10.1038/srep28599 (2016).
